# Reengagement strategies for hepatitis C patients lost to follow-up: A randomized clinical trial

**DOI:** 10.1097/HC9.0000000000000080

**Published:** 2023-05-18

**Authors:** Dalia Morales-Arraez, Alberto Hernández-Bustabad, Cristina Reygosa Castro, Federica Benitez-Zafra, David Nicolás-Pérez, Orestes Crespo, Felicitas Díaz-Flores, Manuel Hernández-Guerra

**Affiliations:** 1 Liver Unit, Canary Islands University Hospital, University of La Laguna, Tenerife, Spain; 2 Administrative Unit, Canary Islands University Hospital, Tenerife, Spain; 3 Central Laboratory Department, Canary Islands University Hospital, Tenerife, Spain; 4 Department of Internal Medicine, University Institute of Biomedical Technologies (CIBICAN), University of La Laguna, Tenerife, Spain

## Abstract

**Background and Aims:**

To achieve the World Health Organization’s goal of eliminating HCV by 2030, reengagement of lost to follow-up cases is mandatory. However, there is lack of evidence concerning the best strategy. Our study evaluated the effectiveness, efficiency, predictive factors, and costs of 2 different strategies.

**Methods:**

We identified patients positive for HCV antibodies without RNA requests from 2005 to 2018. Patients fulfilling trial criteria (NCT04153708) were randomized to (1) phone call or (2) letter of invitation to schedule an appointment, followed by switching strategy.

**Results:**

Three hundred forty-five patients among 1167 lost to follow-up were identified. An analysis of the first 270 randomized patients (72% male, 51±13 y) showed a higher contact rate in the mail than in the phone call strategy (84.5% vs. 50.3%). In the intention-to-treat analysis, no differences were found related to appointment attendance (26.5% vs. 28.5%). Regarding efficiency, 3.1 letters and 8 phone calls were needed to successfully link 1 patient (*p*<0.001) but dropped down to 2.3 phone calls if we only considered the first call attempt (*p*=0.008). Prior specialist’s evaluation and HCV testing in the predirect-acting antiviral era were the only factors associated with no showing up for the appointment. The cost per patient was €621.3 (2.5 quality-adjusted life-years) in the phone call strategy and €611.8 (2.4 quality-adjusted life-years) in the mail letter strategy.

**Conclusions:**

Reengagement of patients with HCV is feasible, and equally effective with similar costs in both strategies. The mail letter was more efficient, except when only 1 phone call was considered. Prior specialist’s evaluation and testing in the predirect-acting antiviral era were factors associated with nonattendance to the appointment.

## INTRODUCTION

HCV infection is a major public health problem and a leading cause of chronic liver disease.^1^ Although most of the patients who failed to undergo interferon-based treatment and those with current follow-ups have been successfully treated with direct-acting antivirals (DAA),^2^ there is still a group of patients without follow-up who are considered to have difficult-to-eliminate HCV.^3^–^5^ Reflex testing, which is 1-step testing using RNA assessment after the detection of positive HCV antibodies, has been shown to reduce the rate of suboptimal diagnosis (ie, those who tested positive for antibodies without RNA investigation) and increases the chances of a referral for treatment.^6^,^7^ In addition, electronic alerts within medical records have been shown to reduce the rate of loss to follow-up.^4^,^8^ However, these strategies have only become broadly implemented in recent years, and many previously diagnosed patients remain lost to follow-up with suboptimal diagnosis or active infection without treatment and at a greater risk of liver fibrosis progression and the development of complications.^9^ A recent and pioneer study carried out in the Netherlands (the REACH project) evaluated patients with suboptimal diagnosis or confirmed active chronic HCV infection who were lost to follow-up and assessed the rate of reengagement by mail, followed by phone in case of nonresponse; the response rate was 43%, and 28% of potential candidates within the health catchment area were eventually reevaluated.^10^ In another study, 29% of patients were recovered by direct phone contact by means of their responsible health care providers.^11^ In a third study conducted in Spain, half of the patients were linked to care after providing a professional multidisciplinary network and contacted by phone call and email several times.^12^ More recently, an active search to retrieve HCV patients lost to follow-up by phone call and reminders by post when telephone contact failed has also proved to be cost-effective.^13^ However, there is a lack of studies evaluating the best reengagement strategy for a microelimination approach, which is important, given the different manpower and time consumption of each different call-back action. Therefore, our aim was to evaluate the effectiveness and efficiency of 2 different strategies (invitation letter by mail or phone call) to reengage patients lost to follow-up, to study predictive factors of response for each evaluated strategy, and analysis of costs of each strategy.

## METHODS

### Study design

Microbiology data files from patients seen between 2005 and 2018 at our tertiary center (Hospital Universitario de Canarias), which serves a population of ~400,000 subjects, were retrospectively reviewed. From all the cases with positive HCV antibodies, we identified those with positive HCV antibodies but without RNA investigation, therefore, unknown RNA.

After excluding deaths from potential candidates for reengagement, we included those fulfilling the following criteria: older than 18 years old, without regular (at least once a year) specialist (internal medicine or hepatology) follow-ups, without HIV coinfection, nondependent people for daily living activities, with enough data available to be contacted (by phone and mail), and those who remain in our health care area. The contact information was gathered from the administrative data records of the patients, which are usually updated after any health-related consultation, including routine blood tests. When the residency address was no longer in our catchment health area, the patient was considered moved to another health area.

Randomization in a 1:1 ratio (ClinicalTrials.gov, Number NCT04153708) was stratified by age and sex, undertaken in blocks of 50 patients, and assigned to any of the following 2 strategies: (1) phone call contact (up to 3 call attempts at different times from inside the hospital) to provide patients with an appointment or (2) mailed invitation letter with a scheduled appointment 10–14 days after receiving the letter. The letters and phone calls were carried out by nonmedical staff (administrative staff). Twenty letters were posted every week, and ~30 calls per day (3 d per week) were performed. In both strategies, the patient was informed about an abnormal laboratory finding and the need to schedule an appointment with a hepatologist for further evaluation. The information given was the same in both strategies, with scarce details in accordance with confidentiality. In addition, in cases where an answering machine or voicemail was encountered, no message was delivered. The letter content and the script used in the phone calls are available as Supplemental Material 1 and 2 (http://links.lww.com/HC9/A174). The letter was registered and returned if the recipient was not found. It included a telephone number, which they could call to change the appointment date. After this first approach, a second chance was provided with a switch of both strategies: patients who were not contacted and patients who did not show up for the appointment were changed to the other strategy, and the same analysis of efficiency and effectiveness was performed.

The number of phone calls needed to contact patients, wrong numbers, nonanswered calls, and returned-to-sender letters were registered. All patients had free access to the health care system called “Seguridad Social” in Spain, without cost for visits, complementary tests, and treatment, if needed.

We registered age, sex, mortality, comorbidity, social support, date of the first positive antibody screen for HCV, previous HBV and HIV tests, transaminases at the time of serology request, history of i.v. or inhaled drug use, and previous specialist evaluation (hepatology or internal medicine department).

Patients who showed up for the appointment were informed about the aims of the study, and informed consent was obtained. Patients who attended the appointment were fully evaluated through a fast-track service, which included elastography (Fibroscan, Echosens, France) and treatment prescription on the same day of the visit; if blood tests were needed for RNA investigation, a second appointment was scheduled within 2 weeks.

Liver fibrosis was assessed using elastography, treatment prescription, and laboratory variables, including RNA results and HBV/HIV status were recorded.

### Effectiveness and efficiency analysis

To compare both strategies, and as a primary aim of the study, we evaluated effectiveness as the rate of patients successfully linked to care (patients who showed up for the appointment with the specialist) and secondary efficiency by considering the number of contacts needed (total number of letters or phone calls) to successfully link 1 patient to care.

### Sample size calculation

A sample size of 172 patients for each strategy was estimated, assuming a 15% increase in effectiveness (from 28% to 43%) of the phone call strategy over the mail strategy (power 80%, alpha error 5%) and a 10% lose in each intervention.

For extraordinary reasons that may have influenced the results because of the nature of the study, an unplanned interim analysis was conducted on March 15th, 2020, 5 days before the national lockdown because of the coronavirus pandemic, which included 270 patients (78% of the planned sample size). One strategy was considered to have a statistically significant difference (*p*<0.001) in the per-protocol (PP) analysis and, mainly because of the aforementioned extraordinary reasons, which may have introduced more bias, the study was stopped.^14^

The switch strategy was postponed after the lockdown was finished and normality arrived at hospital appointments (May 2021).

### Statistics and analysis

Continuous variables were presented as mean (SD) or median (interquartile range) according to the distribution of the data. Categorical variables were presented as absolute frequencies and percentages. The chi-square test was used to compare qualitative variables. Continuous variables were analyzed using the Student *t* test or Mann-Whitney *U* test, according to the distribution of variables was normal. Logistic regression analysis was performed to investigate the independent predictive factors for reengagement.

Intention-to-treat (ITT) and PP analyses were performed. Patients who were successfully contacted were included in the PP analysis. Relative risk (RR) with CIs was calculated for efficiency evaluation.

Statistical significance was set at *p*<0.05. We used SPSS v 25.0. (IBM Corp. Released 2017. IBM SPSS Statistics for Windows, version 25.0, Armonk, NY: IBM Corp.) for statistical analysis.

### Cost-effectiveness analysis

An economic evaluation was performed to estimate the cost of each strategy. It was calculated taking into consideration the actual number of calls, the time dedicated to each call, the number of letters sent, and the time dedicated to sending them. The cost per minute of each call, together with the cost of the administrative staff making the calls was obtained from our Hospital Administrative Department (Supplementary Table 3, http://links.lww.com/HC9/A174).

For cost-effectiveness analysis, a Markov model was designed with a time horizon of 10 years. Cost assessment was performed from the perspective of the Spanish National Health Service, considering direct health costs in both strategies as described.^15^ The costs are expressed in Euros and were updated in 2021 by calculating the Consumer Price Index, estimated by the Spanish National Institute of Statistics (http://www.ine.es/varipc/index.do). The quality-adjusted life-years (QALYs) were taken as the unit to determine the effectiveness of each screening strategy. An annual discount rate of 3% was considered for costs and utilities. The sustained virological response rate of the patients to DAA treatment was assumed to be >90%. The calculation of the incremental cost-effectiveness ratio was performed. A willingness to pay value of €25,000/QALY was chosen as the reference value. A deterministic sensitivity analysis was performed, including a 2-way analysis of the model variables to check their robustness and to identify situations that could change the decision strategy. TreeAge software (TreeAge Software, Williamstown, MA) was used for the construction of the Markov model and the decision analysis.

### Ethical aspects

This study was conducted in accordance with the ethical principles of the Declaration of Helsinki in October 2013 and approved on March 14, 2019, by The Ethics Committee of the Hospital Universitario de Canarias [code CHUC_2019_23 (VHC-ACTIVA)].

## RESULTS

### Patient selection and baseline characteristics

Between 2005 and 2018, 4414 patients were positive for HCV antibodies, of whom 2734 (61.9%) had a negative RNA result or a positive then negative RNA result. After exclusion of 513 (11.6%) patients who died during the study period, 1167 patients were potential candidates for initial reengagement. Finally, 822 (70.4%) were excluded from the study: 713 patients moved to another health area, 53 had HIV coinfection, 35 patients did not have full contact data, 7 were under 18 years old, 12 were linked to care recently pending RNA test results, and 2 patients were dependent people for daily living activities with restricted mobility.

**FIGURE 1:**
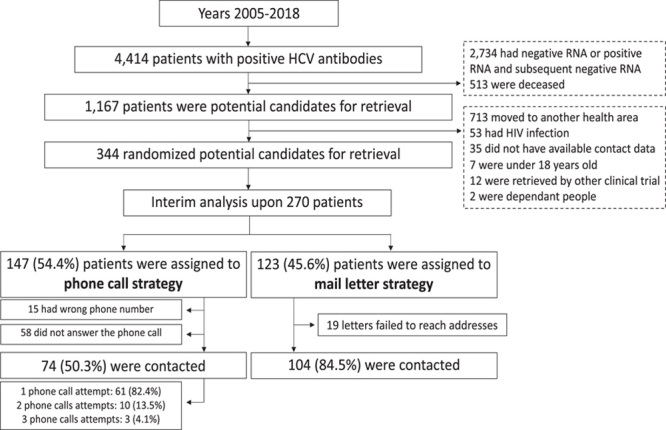
Flow diagram of patient selection and randomization.

Eventually, among the 345 patients fulfilling the criteria to be included in the study, 344 subjects (72.2% male, 51.1±12.9 y) were randomized into the 2 strategies. At the time of the interim analysis, 270 patients were analyzed: 123 patients (45.6%) in the mail strategy and 147 patients (54.4%) in the phone call strategy group (Figure 1). In 22 patients assigned to the mail strategy, the invitation letter was planned to be sent days before the immediate lockdown with an appointment for the following week, and due to timing, were ultimately excluded. There were no differences between the 2 groups in age, sex, comorbidity, history of intravenous or inhaled drug use, social support, time from the first positive HCV serology, testing in the pre-DAA era (before year 2014), years with a positive serology result, abnormal transaminase levels at the time of the serology request, specialist who requested the test, HBV infection, or previous specialist evaluation (Table 1).

**TABLE 1:** Characteristics of randomized patients according to reengagement strategy

	All n=270	Mail letter n=123	Phone call n=147	*p*
Sex, male, n, (%)	195 (72.2)	91 (74)	104 (70.7)	0.587
Age (years, median, IQR)	52.4 (44.4–58.1)	52.4 (45.2–58.9)	52.0 (44.2–57.5)	0.955
Charlson index, ≥2, n (%)	82 (30.4)	38 (29.9)	44 (30.9)	0.895
Poor social support, n (%)	30 (11.2)	14 (11.5)	16 (10.9)	1.000
History of drug use, n (%)	116 (43.1)	53 (43.4)	63 (42.9)	0.877
Time from the first positive anti-HCV test (years, median, IQR)	7 (4–10)	7 (3–11.2)	7 (6–9.7)	0.583
Doctors who requested the serology, n (%)				
Primary care	163 (60.6)	65 (53.3)	98 (66.7)	0.081
Nonprimary care	106 (39.4)	57 (46.7)	49 (33.3)	—
Abnormal transaminases, n (%)	108 (40.1)	48 (39.3)	60 (40.8)	0.901
Previous specialist evaluation, n (%)	40 (14.8)	16 (13)	24 (16.3)	0.494
HCV testing pre-DAA (yes, %)	181 (67)	77 (62.6)	104 (70.7)	0.156
HBV previous request, n (%)	266 (98.9)	121 (99.2)	145 (98.6)	1.000
Positive HBV result, n (%)	4 (1.5)	1 (0.8)	3 (2)	0.649

Abbreviations: DAA, direct-acting antivirals; IQR, interquartile range.

Included patients had a median of 7 (range 4–10) years with a positive HCV serology test, 181 (67%) had the HCV testing serology done before the DAA era, and 15% of patients were previously evaluated by a specialist.

### Effectiveness of both strategies

In the phone call strategy, 15 (10.2%) patients had an incorrect phone number registered in the medical records, and 58 (39.4%) patients did not answer the phone call. Seven (4.8%) patients answered that they did not want to participate in the study. In the mail strategy, 19 (15.4%) letters failed to reach the registered address and were returned. Finally, 74 patients (50.3%) in the phone call strategy and 104 patients (84.5%) in the mail strategy (*p*<0.001) were successfully contacted (had the opportunity to schedule an appointment with the specialist).

**FIGURE 2:**
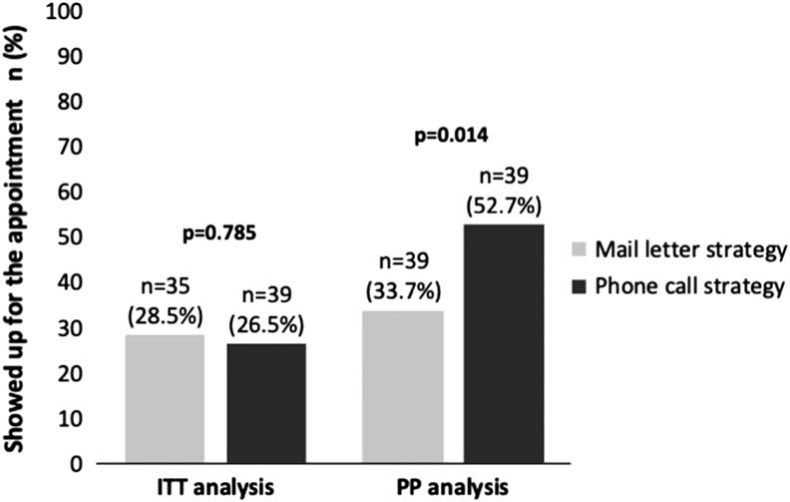
Effectiveness of mail and phone call strategies for reengagement. Abbreviations: ITT, intention to treat, PP, per protocol.

In the ITT analysis (n=270), there were no significant differences between the percentage of patients who showed up for the appointment with the specialist in the phone call strategy compared with the mail strategy (26.5% vs. 28.5%, respectively; *p*=0.785). Instead, in the PP analysis (n=178), the percentage of patients who showed up for an appointment with a specialist was significantly higher in the phone call strategy (52.7% vs. 33.7%, respectively; *p*=0.014). Absolute values are shown in Figure 2.

In the ITT analysis, patients who did not show up for the specialist appointment compared with patients who showed up for the appointment had a higher percentage of patients with a history of injected or inhaled drugs (45.1% vs. 37.8%, respectively; *p*=0.022), higher HCV testing in the pre-DAA era (70.9% vs. 56.8, respectively; *p*=0.027) and a lower rate of prior evaluation by a specialist (11.7% vs. 23%, respectively; *p*=0.033). There were no significant differences between the groups in the PP analysis (Table 2).

**TABLE 2:** Characteristics of patients who attended the specialist appointment compared with patients who did not attend the appointment in ITT and PP analysis

ITT analysis n=270			
	Patients who attended the appointment n=74	Patients who did not attend the appointment n=196	*p*
Sex, male, n, (%)	51 (68.9)	144 (73.5)	0.451
Age (years, median, IQR)	52.4 (45.1–57.3)	52.4 (44.3–58.5)	0.955
Charlson index, ≥2, n (%)	19 (25.7)	63 (32.1)	0.374
Poor social support, n (%)	8 (10.8)	22 (11.3)	0.525
History of drug use, n (%)	28 (37.8)	88 (45.1)	0.022
Time from the first positive anti-HCV test (years, median, IQR)	7 (4–9)	7 (5.2–10)	0.583
Doctors who requested the serology, n (%)			
Primary care	50 (67.6)	113 (57.9)	0.070
Nonprimary care	24 (32.4)	82 (42.1)	—
Abnormal transaminases, n (%)	24 (32.4)	84 (43.1)	0.186
With a previous specialist evaluation, n (%)	17 (23)	23 (11.7)	0.033
HCV testing pre-DAA (yes, %)	42 (56.8)	139 (70.9)	0.027
HBV previous request, n (%)	74 (100)	192 (98.5)	0.564
Positive HBV result, n (%)	1 (1.4)	3 (1.5)	0.558
PP analysis n=178			
	Patients who attended the appointment n=74	Patients who did not attend the appointment n=104	*p*
Sex, male, n (%)	51 (68.9)	72 (69.2)	1.000
Age (years, median, IQR)	52.4 (45.1–57.3)	53.9 (46.5–60.2)	0.879
Charlson index, ≥2, n (%)	19 (25.7)	41 (39.2)	0.076
Poor social support, n (%)	8 (10.8)	8 (7.8)	0.756
History of drug use, n (%)	28 (37.8)	41 (39.8)	0.306
Time from the first positive anti-HCV test (years, median, IQR)	7 (4–9)	7 (3–10.2)	0.952
Doctors who requested the serology, n (%)			
Primary care	50 (67.6)	61 (59.2)	0.184
Nonprimary care	24 (32.4)	42 (40.8)	
Abnormal transaminases, n (%)	24 (32.4)	41 (39.8)	0.604
With a previous specialist evaluation, n (%)	17 (23)	14 (13.5)	0.112
HCV testing pre-DAA (yes, %)	42 (56.8)	65 (62.5)	0.441
HBV previous request, n (%)	74 (100)	102 (99)	1.000
Positive HBV result, n (%)	1 (1.4)	1 (1)	0.679

Abbreviations: DAA, direct-acting antivirals; IQR, interquartile range; ITT, intention to treat; PP, per protocol.

In the ITT analysis, a prior specialist’s evaluation and HCV testing in the pre-DAA era were associated with not showing up for the appointment (Table 3). The results obtained in each strategy separately and in PP analysis are shown in Table 3 and Supplemental Table 2, (http://links.lww.com/HC9/A174), respectively.

**TABLE 3:** Independent predictors for reengagement according to the strategy (ITT analysis)

ITT n=270		
Reengagement		
	OR (95% CI)	*p*
Sex (male vs. female)	1.1 (0.6–2.1)	0.747
Age (≥50 y vs. <50 y)	1.5 (0.8–2.9)	0.265
Charlson index (≥2 vs. <2)	0.5 (0.2–1.1)	0.076
Poor social support (yes vs. no)	0.9 (0.3–2.5)	0.955
History of drug use (yes vs. no)	0.9 (0.6–1.8)	0.950
Time from first positive anti-HCV (≥2 y vs. <2 y)	1.1 (0.7–4.9)	0.202
Abnormal transaminases (yes vs. no)	0.6 (0.3–1)	0.066
Previous specialist evaluation (yes vs. no)	0.4 (0.2–0.8)	0.020
HCV testing pre-DAA (yes vs. no)	0.3 (0.2–0.7)	0.003
Reengagement in the mail strategy n=123		
	OR (95% CI)	*p*
Sex (male vs. female)	1 (0.4–2.7)	0.891
Age (≥50 y vs. <50 y)	1 (0.4–2.7)	0.927
Charlson index (≥2 vs. <2)	0.4 (0.1–1.2)	0.076
Poor social support (yes vs. no)	0.6 (0.1–3)	0.573
History of drug use (yes vs. no)	1 (0.4–2.4)	0955
Time from first positive anti-HCV (≥2 y vs. <2 y)	0.7 (0.2–2.4)	0.764
Abnormal transaminases (yes vs. no)	0.7 (0.3–1.7)	0.353
Previous specialist evaluation (yes vs. no)	2.5 (0.7–8.9)	0.166
HCV testing pre-DAA (yes vs. no)	0.5 (0.2–1.4)	0.178
Reengagement in the phone call strategy n=147		
	OR (95% CI)	*p*
Sex (male vs. female)	1.3 (0.5–3.3)	0.543
Age (≥50 y vs. <50 y)	1.7 (0.6–4.4)	0.287
Charlson index (≥2 vs. <2)	0.8 (0.3–2.4)	0.717
Poor social support (yes vs. no)	0.7 (0.3–6)	0.670
History of drug use (yes vs. no)	1.1 (0.5–2.6)	0.710
Time from first positive anti-HCV (≥2 y vs. <2 y)	3.4 (0.8–14)	0.099
Abnormal transaminases (yes vs. no)	0.6 (0.2–1.4)	0.214
Previous specialist evaluation (yes vs. no)	3 (1–8.1)	0.036
HCV testing pre-DAA (yes vs. no)	0.3 (0.1–0.7)	0.007

Abbreviations: DAA, direct-acting antivirals; ITT, intention-to-treat.

Among patients who showed up for the appointment, there was no significant difference in the waiting time for a specialist appointment in the phone call strategy compared with the mail strategy (median 7 d, interquartile range 7–11 vs. 10 d, interquartile range 10–11, respectively; *p*=0.409).

### Efficiency of both strategies

In the ITT analysis, 3.1 mailed letters and 8 phone calls were necessary to successfully link 1 patient to care with the specialist (RR: 2.53, 95% CI, 1.57–4.07, *p*<0.001), but dropped down to 2.3 phone calls if only the first call attempt was considered as the majority of patients answered and book an appointment with the first call (RR: 0.61, 95% CI, 0.43–0.87, *p*=0.008). When we focused on successfully contacted patients (PP analysis), 2.4 mailed letters and 2.9 phone calls were necessary to successfully book a specialist appointment (RR: 1.25, 95% CI, 0.87–1.79, *p*=0.230).

### Switching strategies

Of 270 patients and after excluding patients who attended the appointment (n=74) and patients who declined participation (n=12), 184 patients remained to be contacted and were switched to the other strategy. Because of the time gap until the switch was performed, 5 (2.7%) patients deceased and 18 (9.8%) received HCV treatment by other means while the study was paused. Finally, 161 (87.5%) patients were included (77% male, 51.4±12.9 y), 82 (51%) in the phone call strategy, and 79 (49%) in the mail letter strategy.

Overall, 44 (53.7%) in the phone call strategy and 62 patients (78.5%) in the mail letter strategy group were successfully contacted (*p*=0.001). Supplemental Figure 1 (http://links.lww.com/HC9/A172) shows the flow chart of the switching strategy.

In the ITT analysis (n=161), there were no significant differences between the proportion of patients who showed up for the appointment in the phone call strategy compared with the mail letter strategy group (22% vs. 11.4%, *p*=0.073). In the PP analysis (n=106), in contrast, the percentage of patients who attended the appointment with the specialist was significantly higher in the phone call strategy group (41% vs. 15%, *p*<0.001). There were no differences between both strategy groups (Supplemental Table 1, http://links.lww.com/HC9/A174).

Regarding efficiency, in the ITT analysis, 8.2 phone calls and 8.8 letters were needed to link to care with the hepatologist (RR: 1.1, 95% CI, 0.7–1.5, *p*=0.517). When only 1 phone call attempt was considered, it dropped down to 4.8 phone calls needed (RR: 1.8, 95% CI, 1.5–3.2, *p*<0.002). When PP analysis was performed, 2 phone calls and 6.8 letters were necessary to book a specialist appointment (RR: 3.4, 95% CI, 2.6–6.2, *p*<0.001).

When all the patients reengaged were considered (n=431) in the analysis after the first reengagement and switch path, similar results were obtained. The phone call strategy was not different to mail letter strategy for patients to show up for the appointment in the ITT analysis (24,9% vs. 21,8%, *p*=0.447); meanwhile, in the PP analysis, the phone call is superior to mail letter (53.3% vs. 26.7%, *p*<0.001).

### Characteristics of reengaged patients

Of the reengaged patients, 101 attended the specialist appointment, of whom 41 (40.6%) had a positive RNA test result. Among patients with active infection, 3 (7.3%) were categorized as F3 and 11 (26.8%) as F4 using elastography. Thirty-eight patients (92.7%) started HCV therapy, and a sustained virological response was tested and documented in 28 (73.7%) patients.

### Cost-effectiveness analysis of each strategy

The direct cost of the phone call strategy was €430.38, whereas the mail letter strategy was €222.6. If we include the switch path in this calculation, the cost of the phone call strategy amounts to €681.08 compared with €360.8 for the mail letter strategy.

**FIGURE 3:**
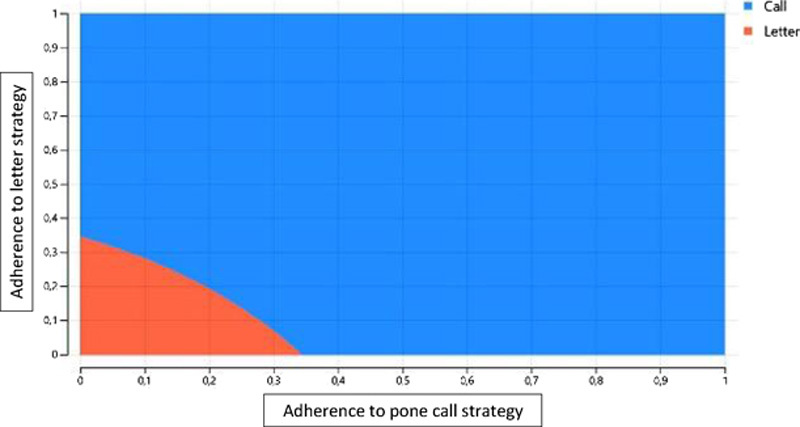
Two-way sensitivity analysis.

The cost per patient who attended the medical visit was €621.3 (2.5 QALY) in the phone call strategy and €611.8 (2.4 QALY) in the mail letter strategy, with an incremental cost-effectiveness ratio of €235/QALY. When the switch is included, the cost of the phone call strategy was €854.2 (1.9 QALY) and €650 (1.1 QALY), with an incremental cost-effectiveness ratio of €259/QALY. Figure 3 shows the relation between both the strategies’ adherence (successfully reengaged patients), where a high adherence would support the phone call strategy; meanwhile, it seems to be more cost-effective the mail letter if a low adherence is presumed. Supplemental Figure 2 (http://links.lww.com/HC9/A173) shows the results when the switch is included, where the greater the number of contacts, the greater the success of the strategy.

## DISCUSSION

In this randomized clinical trial, we investigated the effectiveness and efficiency of 2 different strategies for reengagement in specialist care for HCV patients lost to follow-up. In our setting, both the strategies were feasible and similar in terms of effectiveness; meanwhile, the mail letter strategy seemed to be more efficient, except if only 1 call attempt was considered, in which case, the phone call was superior. Prior specialist’s evaluation and being tested for HCV in the pre-DAA era were the factors associated with not showing up for the appointment. Both strategies were similar in costs. Patients with suboptimal diagnosis, that is, with a positive HCV antibody with unknown RNA, and those with active infection lost to follow-up, were considered a priority group to be linked to care in the microelimination plans^16^ mainly because these patients have a high probability of active infection and are at risk of liver fibrosis progression^9^ and late presentation.^17^ It is important to emphasize that in our cohort of 13 years of testing, nearly one fourth of the patients with positive serology for HCV remained without full evaluation or treatment for a median of 7 years with positive HCV antibodies, which is consistent with other studies.^16^ Some might think that these patients are lost to follow-up because they are likely to be difficult to treat; however, this group of patients is characterized as being young male without comorbidity, with a history of drug use, and having abnormal transaminases without previous specialist evaluation. This is the rationale for focusing the study on this population with suboptimal diagnosis, which is often largely forgotten, despite a non-negligible rate of 41% positivity for RNA-HCV in our cohort. This rate and profile of patients^18^ should be considered in addition to those with RNA-positive testing lost to follow-up when implementing reengagement plans if attempting to reach World Health Organization goals by 2030. It is worth noting that patients with positive RNA may have a higher interest in attending the appointments in reengagement plans. In fact, in the REACH project,^10^ 42 out of 47 traced individuals turned out to have a persistent chronic HCV infection, and 82.4% of lost to follow-up patients attended in another interventional study were RNA positive.^12^

The first reengagement attempt implemented to rescue patients lost to follow-up using laboratory records was conducted in the Netherlands, where patients were invited to participate by a mailed letter.^19^ However, mail succeeded in only half of the patients lost to follow-up. Several obstacles have been considered, such as the fact that letters do not fully respect the patient’s privacy, the impersonal nature of the letters makes it very difficult to detect the tone of the sender, which may influence its success rate, they do not consider patients’ preferences for a date, or more importantly, do not reach the patient due to incorrect residency information. For these reasons, other strategies, such as direct phone contact with the patient, have been evaluated and are arguably a more attractive and feasible approach for contacting patients. In a recently published study, the rate of reengaged patients using phone calls was 63%^20^; however, several strategies, such as automatic reminders in the electronic clinical records, were implemented.^4^ To the best of our knowledge, this is the first randomized trial to evaluate 2 different approaches within a reengagement plan to link patients lost to follow-up.

In our study, the number of patients contacted by mail was 34% higher than that by phone calls. However, in the phone call strategy, half of the patients were successfully contacted and scheduled for an appointment, a rate similar to that in the REACH study considering the mail strategy. However, in terms of contacting potential cases, both the strategies are insufficient and far from perfect if patient reengagement is the main objective.

The high number of eligible patients for reengagement that were excluded from the study because of the change in residency or no contact data available since the HCV diagnostic test date recorded (overall 64%) was the main limiting factor in the development of successful reengagement strategies. In this regard, automatic electronic alerts addressed to the primary care physician for a referral to specialist cases with HCV antibodies could be a better way to contact this group of difficult-to-reach patients.^8^ In addition, microbiology and administrative departments should be permitted to share data regarding HCV infection status and exchange updated personal information providing higher chances of contacting patients, although overall results, despite the coordinated efforts, may result suboptimal with the current contact strategy.^21^

Our findings support that the mail strategy is more efficient than the phone call strategy with a 3.1 ratio of contact attempts, except when only 1 call attempt is considered in the analysis, which then favors the phone call strategy. In our study, to improve the chances of contact, up to 3 different attempts at different times were planned. The use of answering machines could increase efficiency. Nevertheless, because of confidentiality reasons, their use was not permitted.

The phone call strategy nearly doubled the percentage of patients who attended the appointment after contact was successfully established (PP analysis 52.7% vs. 33.7%). Therefore, phone calls were more effective than letters when contact was established; however, in the ITT analysis among randomized patients, there were no differences (26.5% vs. 28.5%). It is still unclear whether mobile text messages would be more effective than phone calls, as there is evidence suggesting that mobile messages and phone calls have similar efficacy in promoting attendance at health care appointments.^22^ Patients in the phone call strategy had flexibility in scheduling appointments according to their preferences, which may be an intrinsic advantage of the phone strategy; for this reason, a better-accepted strategy by patients increases adherence to the appointment. However, among patients who eventually attended the appointment, there was no difference in time to be scheduled between the phone and mail strategy, suggesting that this potential advantage did not improve compliance. In addition, a 3-day difference may not be of sufficient relevance to alter the results.

One recent study has demonstrated that reengagement is cost saving for the public health system because of the estimated reductions in liver disease complications and mortality, being able to link to care 26% of the candidate patients, which is quite similar to our global results (27.4%).^13^ In our study, attending to the strategy and direct health costs derived from lost to follow-up, treatment, and follow-up of these patients, mail letter has shown to be the most cost-effective intervention when a low adherence to these strategies is presumed, whereas switch to the phone call as adherence to the program increases.^23^

With both the strategies, patients could perceive a medical confidentiality breach. However, the basic principles of medical ethics were considered,^24^ and it could be disregarded from a public health perspective,^25^ given the advantages that early treatment of HCV confers in preventing disease progression^26^ and cost-effectiveness.^27^ In addition, regulators in our country have been clear about this ethical point and favorably encourage this reengagement approach.^28^ In any case, both the strategies seem to exceed possible risks, as almost half of the reengaged patients confirmed having an active infection, of whom 30% had at least advanced fibrosis, and over 90% of patients eventually received treatment in agreement with the other reengagement study performed in our country.^12^

The factors associated with unsuccessful reengagement were previous evaluation by any specialist and HCV-antibody detection in the pre-DAA era. None of the reengaged patients were previously treated, so it is tempting to speculate that patients who were lost to follow-up after being evaluated by a specialist, keep in mind the complexity and adverse effects of previous treatment regimens or the lack of compliance perceived by the physicians. In this regard, news media information about DAAs is relevant and could improve linkage to care, as shown in this study and by our group.^29^ On the other hand, sex, age, comorbidity, abnormal liver tests, time from the first positive HCV test or specialist who requested the test, history of intravenous drug use, or poor social support did not influence the efficacy of both evaluated strategies. This contrasts with evidence from referral-to-care studies suggesting that people with a history of intravenous drug use are a well-known difficult-to-link-to-care population.^16^ Since 2017, we have had a close relationship of collaboration with drug addiction centers in our health care area, firmly committed to the goals of diagnosing, referral, and monitoring treatment of patients with HCV infection on methadone maintenance program^30^ and we cannot rule out that these centers may have influenced and encouraged patients to attend the appointments.

Imprison and homeless population are usually excluded from reengagement plans^10^,^12^ to be approached in more targeted initiatives to reach homeless patients^31^ patients at penitentiary institutions by telemedicine, which is a support tool that has proven to be successful^32^ and a more efficient strategy than usual clinical practice.^33^ This study has some limitations. First, <30% of candidates for reengagement were eligible for the study, given the strict inclusion criteria, and our results may not apply to real-life settings. Second, <90% of patients with active infection who attended the appointment started treatment. This rate exceeded expectations, and our fast-track service may have influenced this result, so reengagement plans might have to include simplified circuits of care. Third, we used contact data available from medical records, which may not have been recently updated, affecting both strategies. Fourth, the study did not include patients with positive RNA lost to follow-up, and the response rate of this group of patients remains to be studied. RNA-positive cases are relevant and must be included in any reengagement plan. However, we decided to exclude known RNA-positive cases from the trial as most of these patients have previously been contacted after DAA approval and urgent implementation of projects given the high risk of fibrosis progression of these patients, and to stick to the trial strict inclusion criteria to minimize any bias recruiting patients with probably a higher interest in attending the appointments. In fact, in the REACH project^10^ 42 out of 47 traced individuals turned out to have a persistent chronic HCV infection, and 82.4% of lost to follow-up patients attended in another interventional study were RNA positive.^12^ Fifth, the finding that not being previously evaluated by a specialist is associated with successful reengagement cannot be extended outside this cohort since it may be influenced by the fact that those correctly followed up and treated by a specialist are not included in our database. Finally, we decided to stop the clinical trial after achieving statistical differences in the PP analysis after the unplanned interim analysis. The current coronavirus pandemic could have influenced the results because all scientific guidelines recommended the reduction or suspension of nonurgent face-to-face appointments to minimize the expected increased care burden.^34^ However, the interim analysis was performed with almost 80% of the planned sample size enrolled, and if the study was to continue, it is unlikely that the results would be significantly different. In addition, a switch strategy round was performed after the pandemic lockdown with similar results.

In conclusion, the phone call and mail strategies are feasible for the reengagement of HCV patients lost to follow-up. Both the strategies were similar in effectiveness and costs, whereas the mail strategy was more efficient, except when only 1 phone call attempt was considered. This pragmatic study may guide other institutions to implement a systematic strategy to reengage care for HCV-infected patients and may contribute to achieving HCV microelimination and get us closer to meeting the World Health Organization elimination goals.

## Supplementary Material

SUPPLEMENTARY MATERIAL
