# Supplementary figures and images for: Reengagement strategies for hepatitis C patients lost to follow-up: A randomized clinical trial
Source: Hepatol Commun. 2023 May 18;7(6):e0080. doi: 10.1097/HC9.0000000000000080 (PMC10538908; doi:10.1097/HC9.0000000000000080)

**Supplementary figure 1.** Flow diagram of switch strategy.

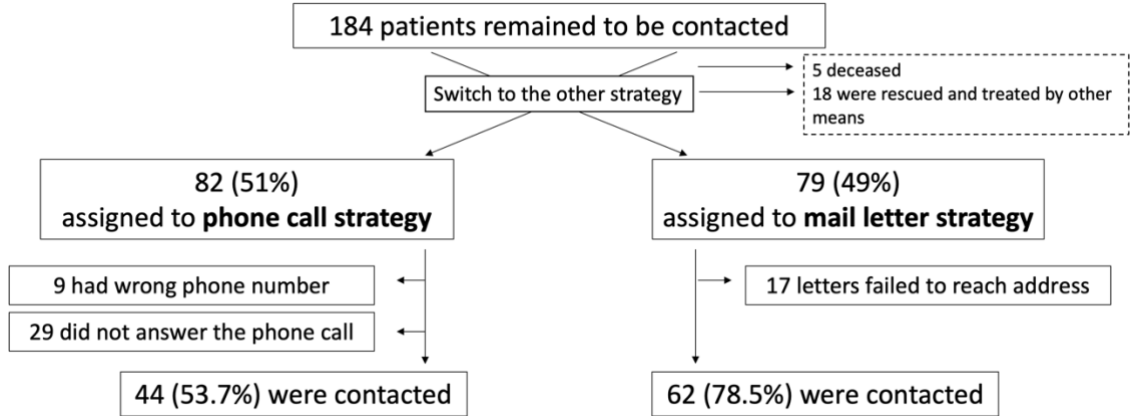

Supplement: SUPPLEMENTARY MATERIAL [file hc9-7-e0080-s001.pdf]

**Supplementary figure 2.** Two-way sensitivity analysis including Switch.

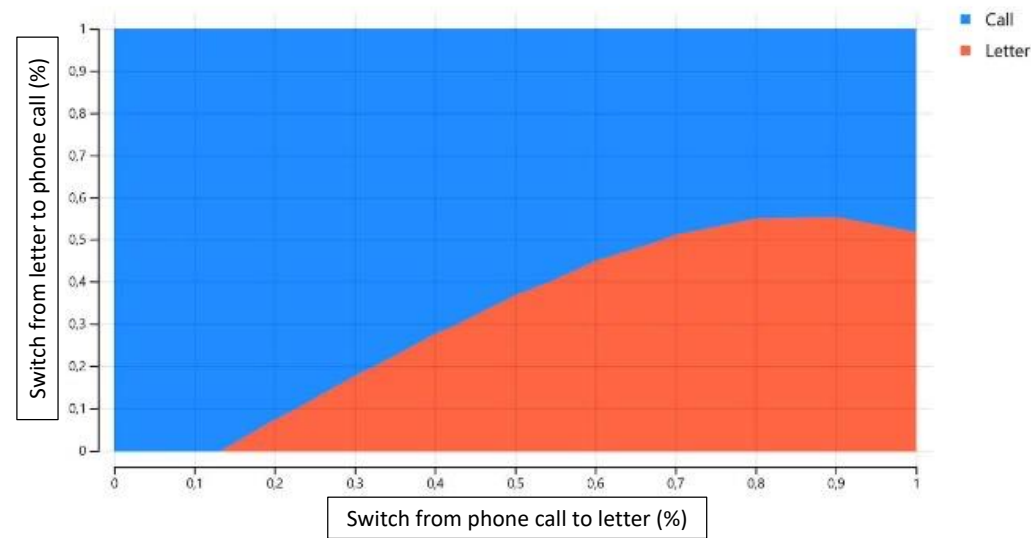

Supplement: SUPPLEMENTARY MATERIAL [file hc9-7-e0080-s002.pdf]
