# Supplementary material for: Reengagement strategies for hepatitis C patients lost to follow-up: A randomized clinical trial
Source: Hepatol Commun. 2023 May 18;7(6):e0080. doi: 10.1097/HC9.0000000000000080 (PMC10538908; doi:10.1097/HC9.0000000000000080)
Supplement: SUPPLEMENTARY MATERIAL [file hc9-7-e0080-s003.docx]

**Supplementary table 1.** Characteristics of patients who attended the specialist appointment compared to patients who did not attend the appointment in ITT and PP analysis in switch strategy

| **ITT analysis**  **n=161** | | | |
| --- | --- | --- | --- |
|  | **Patients who attended the appointment**  **n=27** | **Patients who did not attend the appointment**  **n=134** | **p-value** |
| Gender (male, n, %) | 23 (85.2) | 101 (75.4) | 0.269 |
| Age (years, median, IQR) | 53.5 (42.3-61-6) | 52.3 (44.3-57.3) | 0.709 |
| Charlson index (≥2, n, %) | 11 (40.7) | 37 (27.6) | 0.174 |
| Poor social support (n, %) | 2 (7.4) | 17 (12.8) | 0.431 |
| History of drug use (n, %) | 11 (40.7) | 67 (50.4) | 0.403 |
| Time from the first positive anti-HCV test (years, median, IQR) | 9 (5-9) | 9 (5-9) | 0.186 |
| Abnormal transaminases (n, %) | 11 (40.7) | 61 (45.9) | 0.626 |
| With a previous specialist evaluation (n, %) | 1 (3.7) | 13 (9.7) | 0.313 |
| HBV previous request (n, %) | 74 (100) | 192 (98.5) | 0.564 |
| Positive HBV result (n, %) | 1 (1.4) | 3 (1.5) | 0.558 |
| **PP analysis**  **n=94** | | | |
|  | **Patients who attended the appointment**  **n=27** | **Patients who did not attend the appointment**  **n=67** | **p-value** |
| Gender (male, n, %) | 23 (86) | 54 (80.6) | 0.601 |
| Age (years, median, IQR) | 52 (40-59.6) | 53.4 (43.8-56.8) | 0.854 |
| Charlson index (≥2, n, %) | 11 (40.7) | 16 (24) | 0.102 |
| Poor social support (n, %) | 2 (7.4) | 10 (15) | 0.323 |
| History of drug use (n, %) | 11 (41) | 35 (52) | 0.306 |
| Time from the first positive anti-HCV test (years, median, IQR) | 7 (5-9) | 8 (4.8-9) | 0.213 |
| Abnormal transaminases (n, %) | 11 (40.7) | 31 (46.3) | 0.604 |
| With a previous specialist evaluation (n, %) | 4 (27) | 12 (57) | 0.070 |
| HBV previous request (n, %) | 26 (96) | 66 (99) | 1.000 |
| Positive HBV result (n, %) | 0 | 1 (1.5) | 0.655 |

ITT: intention to treat, PP: per protocol, IQR: interquartile range, HCV: hepatitis C virus, PP: per protocol

**Supplementary table 2.** Independent predictors for re-engagement according to strategy (PP analysis).

| **PP**  **n=178** | | |
| --- | --- | --- |
| **Re-engagement** | | |
|  | **OR (95%CI)** | **p-value** |
| Gender (male vs. female) | 1 (0.5–2.1) | 0.895 |
| Age (≥ 50 years vs. < 50 years) | 1 (0.5–2.2) | 0.965 |
| Charlson index (≥2 vs. <2) | 0.5 (0.2–1.2) | 0.132 |
| Poor social support (yes vs. no) | 0.8 (0.3–2.7) | 0.789 |
| History of drug use (yes vs. no) | 0.8 (0.4–1.6) | 0.639 |
| Time from first positive anti-HCV (≥2 years vs. <2 years) | 1.3 (0.5–3.2) | 0.522 |
| Abnormal transaminases (yes vs. no) | 0.8 (0.4–1.5) | 0.428 |
| Previous specialist evaluation (yes vs. no) | 0.4 (0.2–1) | 0.054 |
| **Re-engagement** **in the mail strategy**  **n=104** | | |
|  | **OR (95%CI)** | **p-value** |
| Gender (male vs. female) | 1 (0.4–2.7) | 0.978 |
| Age (≥ 50 years vs. < 50 years) | 1.1 (0.4–2.6) | 0.819 |
| Charlson index (≥2 vs. <2) | 0.4 (0.1–1.5) | 0.202 |
| Poor social support (yes vs. no) | 0.6 (0.1–2.7) | 0.512 |
| History of drug use (yes vs. no) | 0.9 (0.4–2.2) | 0.880 |
| Time from first positive anti-HCV (≥2 years vs. <2 years) | 1.2 (0.4–4) | 0.750 |
| Abnormal transaminases (yes vs. no) | 0.7 (0.3–1.7) | 0.403 |
| Previous specialist evaluation (yes vs. no) | 0.3 (0.1–1.2) | 0.088 |
| **Re-engagement** **in the phone call strategy**  **n=74** | | |
|  | **OR (95%CI)** | **p-value** |
| Gender (male vs. female) | 1.1 (0.3–3.5) | 0.879 |
| Age (≥ 50 years vs. < 50 years) | 1.2 (0.3–4.4) | 0.744 |
| Charlson index (≥ 2 vs. < 2) | 0.6 (0.2–2.1) | 0.429 |
| Poor social support (yes vs. no) | - | 0.999 |
| History of drug use (yes vs. no) | 0.7 (0.2–2.5) | 0.611 |
| Time from first positive anti-HCV (≥ 2 years vs. < 2 years) | 3.2 (0.7–14) | 0.130 |
| Abnormal transaminases (yes vs. no) | 0.9 (0.3–2.7) | 0.882 |
| Previous specialist evaluation (yes vs. no) | 0.6 (0.2–2.4) | 0.523 |

PP: per protocol, OR: odds ratio, CI: confidence interval, HCV: hepatitis C virus

**Supplementary table 3. Direct costs associated to each strategy.**

| **General costs** |  |
| --- | --- |
| - Phone call | €1.08 per minute |
| - Mail letter | €1.16 per letter |
| - Administrative staff | €14.8 per hour |
|  |  |
| **Number of phone calls (1^st^ stage)** | 279 |
| **Number of phone calls (switch)** | 158 |
| **Time estimated per phone call (minutes)** | 3 |
| **Time estimated per missed call (minutes)** | 0.5 |
|  |  |
| **Number of letters sent (1^st^ stage)** | 123 |
| **Number of letters sent (switch)** | 79 |
|  |  |
| **Total cost of phone call strategy (including switch)** | €681.1 |
| **Total cost of mail letter strategy (including switch)** | €360.8 |

**Supplementary figure 1.** Flow diagram of switch strategy.

**Supplementary figure 2.** Two-way sensitivity analysis including Switch.

**Supplementary material 1.** Information given to the administrative staff for calling in phone call strategy.

- Introduction:

*“Good morning, this is “administrative NAME”, I am calling from the Gastroenterology and Hepatology Department of the Hospital Universitario de Canarias. Are you “patient's NAME”?”*

If positive answer, you can continue.

- If it is not the patient:

*“Would it be possible to put me through to “patient's NAME”? A contact telephone number where I can get in touch with him/her?”*

Important: do not leave a message about the appointment unless they confirm that it is a close family member (wife or children) or cohabitant and we will try to agree to repeat the call at another time.

- If it is the patient, explain reason for calling:

*"We are calling you to give you an appointment in consultations since we have detected that you have been diagnosed in the Central Laboratory of the Hospital Universitario de Canarias of a process that may require a more detailed study to assess whether this has been resolved or requires some kind of treatment or follow-up."*

- In case of requesting more information a:

*“It is not possible to give you that information for data protection reasons, but the doctor will give you in detail all the information in the consultation.”*

Important: for data protection reasons we cannot tell anything that has to do with the pathology we are treating (HCV). We must convey reassurance, but at the same time be assertive about the need for the consultation: "It is recommended that you do not miss the appointment", for example.

**Supplementary material 2.** Letter sent to the patient in mail letter strategy.

*Dear Sir/Madam: “NAME AND SURNAME”*

*We are contacting you because we have detected that you have been diagnosed in the Central Laboratory of the Hospital Universitario de Canarias of a process that may require a more detailed study to assess whether it has been resolved or requires some kind of treatment or follow-up.*

*For this reason, we summon you to go to the Gastroenterology and Hepatology Department of the Hospital Universitario de Canarias from 9:00 am to 1:00 pm on the “DATE”. In case you are unable to attend, please contact us by phone at “PHONE” from Monday to Friday from 09:00am-1:00pm.*

*For confidentiality reasons we will inform you of the process that will be assessed in the scheduled consultation.*

*As always, our goal is to improve your health and quality of life.*

*Best regards.*
